# Supplementary material for: A Model for Consumer Acceptance of Insect-Based Dog Foods among Adult UK Dog Owners
Source: Animals (Basel). 2024 Mar 27;14(7):1021. doi: 10.3390/ani14071021 (PMC11010811; doi:10.3390/ani14071021)
Supplement: Supplementary file 1 [file animals-14-01021-s001.zip › animals-2919301-supplementary.pdf]

Table S1- Mean and Standard Deviation for each Construct

Constructs	Mean	Standard Deviation
Food Neophobia (FN)	2.843	1.241
Attitudes towards Uses of Animals (AUA)	3.780	1.239
Food Preference- Animal Welfare (FPW)	4.104	0.942
Food Preference- Environment (FPE)	3.726	0.981
Food Preference- Health (FPH)	4.213	0.881
Disgust (AICD)	2.836	1.294
Beliefs regarding Insect Sentience (AICS)	3.359	1.032
Perceptions of Benefits (AICB)	3.732	0.826
Perceptions of Risks (AICR)	2.661	0.755
Perceived Behavioural Control (PBC)	4.118	0.967
Perceived Barriers (PB)	3.448	0.984
Social Norms (SN)	2.900	1.147
Attitudes towards Insect-Based Dog Food (AIDF)	3.436	0.806
Intention to Try (ITT)	3.537	1.133
Intention to Buy (ITB)	3.415	1.093

Table S2- Survey

**Question**	**Answer options**
Eligibility Questions- Please answer the following eligibility questions	
Are you aged 18 years or over?	Yes<br>No
Are you resident in the United Kingdom?	Yes<br>No
Do you own or are you responsible for the care of one or more dogs?	Yes<br>No
Please select the option below which best represents your level of agreement with each of the following statements:	
I am constantly sampling new and different foods*	Strongly Disagree<br>Disagree<br>Neither Agree nor Disagree<br>Agree<br>Strongly Agree
I do not trust new foods	Strongly Disagree<br>Disagree<br>Neither Agree nor Disagree

Agree
Strongly Agree

If I do not know what is in a food I will not eat it
Strongly Disagree
Disagree
Neither Agree nor Disagree
Agree
Strongly Agree

I am afraid to eat things I have never had before
Strongly Disagree
Disagree
Neither Agree nor Disagree
Agree
Strongly Agree

I will eat almost anything*
Strongly Disagree
Disagree
Neither Agree nor Disagree
Agree
Strongly Agree

Please select the option below which best represents your level of agreement with each of the following statements:

It is morally wrong to hunt wild animals for sport
Strongly Disagree
Disagree
Neither Agree nor Disagree
Agree
Strongly Agree

I do not think that there is anything wrong with using animals in medical research*
Strongly Disagree
Disagree
Neither Agree nor Disagree
Agree
Strongly Agree

I think it is perfectly acceptable for cattle and pigs to be raised for human consumption*
Strongly Disagree
Disagree
Neither Agree nor Disagree
Agree
Strongly Agree

The slaughter of whales and dolphins should be immediately stopped even if it means some people will be put out of work
Strongly Disagree
Disagree
Neither Agree nor Disagree
Agree
Strongly Agree

I sometimes get upset when I see wild animals in cages at zoos
Strongly Disagree
Disagree
Neither Agree nor Disagree
Agree
Strongly Agree

Please select the option below which best represents your level of agreement with each of the following statements:

It is important to me that the food I buy is not normally produced by hurting animals
Strongly Disagree
Disagree
Neither Agree nor Disagree

	Agree
	Strongly Agree
It is important to me that the food I buy has been produced in a way that respects animal rights	Strongly Disagree<br>Disagree<br>Neither Agree nor Disagree<br>Agree<br>Strongly Agree
I think that more regulation is needed on how to treat animals in agriculture	Strongly Disagree<br>Disagree<br>Neither Agree nor Disagree<br>Agree<br>Strongly Agree

Please select the option below which best represents your level of agreement with each of the following statements:

My food purchasing habits are affected by my concern for the environment	Strongly Disagree<br>Disagree<br>Neither Agree nor Disagree<br>Agree<br>Strongly Agree
I am worried about wasting the planet's resources	Strongly Disagree<br>Disagree<br>Neither Agree nor Disagree<br>Agree<br>Strongly Agree
I consider the potential environmental impact of my actions when I make many of my decisions	Strongly Disagree<br>Disagree<br>Neither Agree nor Disagree<br>Agree<br>Strongly Agree

.

Please select the option below which best represents your level of agreement with each of the following statements:

The healthiness of my dog's food has little impact on my choice of food for them*	Strongly Disagree<br>Disagree<br>Neither Agree nor Disagree<br>Agree<br>Strongly Agree
I am very particular about the healthiness of the food my dog eats	Strongly Disagree<br>Disagree<br>Neither Agree nor Disagree<br>Agree<br>Strongly Agree
I feed my dog what he/she likes and do not worry much about the healthiness of the food*	Strongly Disagree<br>Disagree<br>Neither Agree nor Disagree<br>Agree<br>Strongly Agree
It is important to me that the food my dog eats is nutritious	Strongly Disagree<br>Disagree<br>Neither Agree nor Disagree

	Agree
	Strongly Agree

Please select the option below which best represents your level of agreement with each of the following statements:

The idea of eating insects makes me feel nauseous	Strongly Disagree<br>Disagree<br>Neither Agree nor Disagree<br>Agree<br>Strongly Agree8
I am offended by the idea of eating insects	Strongly Disagree<br>Disagree<br>Neither Agree nor Disagree<br>Agree<br>Strongly Agree
Eating insects is disgusting	Strongly Disagree<br>Disagree<br>Neither Agree nor Disagree<br>Agree<br>Strongly Agree

Please select the option below which best represents your level of agreement with each of the following statements:

I think that insects are capable of feeling pain	Strongly Disagree<br>Disagree<br>Neither Agree nor Disagree<br>Agree<br>Strongly Agree
I think that Insects are capable of experiencing suffering	Strongly Disagree<br>Disagree<br>Neither Agree nor Disagree<br>Agree<br>Strongly Agree
Insects have consciousness	Strongly Disagree<br>Disagree<br>Neither Agree nor Disagree<br>Agree<br>Strongly Agree
Insects have rights	Strongly Disagree<br>Disagree<br>Neither Agree nor Disagree<br>Agree<br>Strongly Agree

Please select the option below which best represents your level of agreement with each of the following statements:

Rearing insects for food generates less pollution and greenhouse gas than rearing conventional livestock	Strongly Disagree<br>Disagree<br>Neither Agree nor Disagree<br>Agree<br>Strongly Agree

Rearing insects as food is more efficient and requires fewer resources than rearing conventional livestock	Strongly Disagree<br>Disagree<br>Neither Agree nor Disagree<br>Agree<br>Strongly Agree
Rearing insects for food requires much less space than rearing conventional livestock	Strongly Disagree<br>Disagree<br>Neither Agree nor Disagree<br>Agree<br>Strongly Agree
Insects contain high levels of high quality animal protein	Strongly Disagree<br>Disagree<br>Neither Agree nor Disagree<br>Agree<br>Strongly Agree
Insects are highly nutritious	Strongly Disagree<br>Disagree<br>Neither Agree nor Disagree<br>Agree<br>Strongly Agree

Please select the option below which best represents your level of agreement with each of the following statements:

Insects contain harmful toxins	Strongly Disagree<br>Disagree<br>Neither Agree nor Disagree<br>Agree<br>Strongly Agree
Eating insects would expose me to harmful chemicals and insecticides	Strongly Disagree<br>Disagree<br>Neither Agree nor Disagree<br>Agree<br>Strongly Agree
Eating insects will increase risk of infectious disease	Strongly Disagree<br>Disagree<br>Neither Agree nor Disagree<br>Agree<br>Strongly Agree

The remaining questions in this survey will refer to "insect-based dog food". Insect-based dog food is an innovative dog food with insects as the core ingredient. Seven species of insect are currently authorized for use in pet food in the EU and the UK of which the most commonly used are: house crickets, yellow mealworms and black soldier flies. Companies have already successfully launched insect-based dog food products in stores and online.

Please select the option below which best represents your level of agreement with each of the following statements:

The decision to feed my dog products containing insect-based ingredients in the next month is under my complete control	Strongly Disagree<br>Disagree<br>Neither Agree nor Disagree<br>Agree

	Strongly Agree
Feeding products containing insect based ingredients to my dog in the next month is completely up to me	Strongly Disagree<br>Disagree<br>Neither Agree nor Disagree<br>Agree<br>Strongly Agree

Please select the option below which best represents your level of agreement with each of the following statements:

Insect-based dog foods are more expensive	Strongly Disagree<br>Disagree<br>Neither Agree nor Disagree<br>Agree<br>Strongly Agree
Insect-based dog foods are not available in my local shops	Strongly Disagree<br>Disagree<br>Neither Agree nor Disagree<br>Agree<br>Strongly Agree
I have little access to information about insect-based dog foods	Strongly Disagree<br>Disagree<br>Neither Agree nor Disagree<br>Agree<br>Strongly Agree

Please select the option below which best represents your level of agreement with each of the following statements:

I would be more likely to feed my dog insect-based dog foods if recommended by my family	Strongly Disagree<br>Disagree<br>Neither Agree nor Disagree<br>Agree<br>Strongly Agree
I would be more likely to feed my dog insect-based dog foods if recommended by my friends	Strongly Disagree<br>Disagree<br>Neither Agree nor Disagree<br>Agree<br>Strongly Agree
I would be more likely to feed my dog insect-based dog foods if recommended by my colleagues/peers	Strongly Disagree<br>Disagree<br>Neither Agree nor Disagree<br>Agree<br>Strongly Agree

Please select the option below which best represents your level of agreement with each of the following statements:

Insect-based dog food is healthy	Strongly Disagree<br>Disagree<br>Neither Agree nor Disagree<br>Agree<br>Strongly Agree
Insect-based dog food is safe for dogs to eat	Strongly Disagree

	Disagree
	Neither Agree nor Disagree
	Agree
	Strongly Agree
Insect-based dog food is more sustainable than most ordinary dog foods	Strongly Disagree
	Disagree
	Neither Agree nor Disagree
	Agree
	Strongly Agree
Insect-based dog food is better for animal welfare compared to most ordinary dog foods	Strongly Disagree
	Disagree
	Neither Agree nor Disagree
	Agree
	Strongly Agree

Please select the option below which best represents your level of agreement with each of the following statements:

I would be willing to try feeding insect based food to my dog if it were widely available in stores	Strongly Disagree
	Disagree
	Neither Agree nor Disagree
	Agree
	Strongly Agree
I would be willing to try feeding insect based food to my dog if it were recommended by vets	Strongly Disagree
	Disagree
	Neither Agree nor Disagree
	Agree
	Strongly Agree
I would be willing to try feeding insect based food to my dog if free samples were available	Strongly Disagree
	Disagree
	Neither Agree nor Disagree
	Agree
	Strongly Agree

Please select the option below which best represents your level of agreement with each of the following statements:

I would buy insect- based dog food if it were produced in a more environmentally- friendly way than ordinary dog food	Strongly Disagree
	Disagree
	Neither Agree nor Disagree
	Agree
	Strongly Agree
I would buy insect- based dog food if it had more micronutrients than ordinary dog food	Strongly Disagree
	Disagree
	Neither Agree nor Disagree
	Agree
	Strongly Agree
I would buy insect- based dog food if it were as accessible as ordinary dog food	Strongly Disagree
	Disagree
	Neither Agree nor Disagree
	Agree
	Strongly Agree

Supplementary Materials

I would buy insect- based dog food if it had a similar look and texture as ordinary dog food	Strongly Disagree<br>Disagree<br>Neither Agree nor Disagree<br>Agree<br>Strongly Agree
I would buy insect- based dog food if it were from a renowned brand	Strongly Disagree<br>Disagree<br>Neither Agree nor Disagree<br>Agree<br>Strongly Agree
I would buy insect- based dog food if it were cheaper than ordinary dog food	Strongly Disagree<br>Disagree<br>Neither Agree nor Disagree<br>Agree<br>Strongly Agree
Demographic Questions<br>Finally, please answer the following demographic questions	
What is your gender?	Male<br>Female<br>Other<br>Prefer not to say
What is your age?	18-30<br>31-45<br>46-64<br>65 and over
How would you describe your dietary preference?	Omnivore (I eat most animal products including meat, fish, seafood and/or dairy)<br>Semi-vegetarian/ Flexitarian (I am cutting back on meat but not avoiding it completely)<br>Full-time Vegetarian (I do not eat meat but am still eating other animal products)<br>Vegan (I do not eat any animal products)

Statements marked * were reverse scored
